# Influence of individual and partner preferences on joint condom use during vaginal sex among young people in the Netherlands: a statistical modelling study

**DOI:** 10.1136/bmjph-2025-003606

**Published:** 2026-10-07

**Authors:** Alcira de Vries, Disa Hansson, John B F de Wit, Chantal den Daas, Janneke C M Heijne

**Affiliations:** 1 Centre for Infectious Disease Control, National Institute for Public Health and the Environment, Bilthoven, The Netherlands; 2 European Centre for Disease Prevention and Control, Solna, Sweden; 3 Utrecht University, Utrecht, The Netherlands; 4 University of Aberdeen, Aberdeen, Scotland, UK; 5 Public Health Service of Amsterdam, Amsterdam, The Netherlands; 6 Amsterdam Public Health Research Institute, Amsterdam, The Netherlands

**Keywords:** Disease Transmission, Infectious, Sexually Transmitted Diseases, statistics and numerical data, Adolescent

## Abstract

**Introduction:**

Condom use during penile–vaginal sex is decreasing among youth. Little research exists on the potential influence of both partners on joint condom use, especially including individual psychosocial factors and sex. Condom dispositions are individuals’ behavioural tendencies to use a condom or not. Using statistical models, we aimed to understand how psychosocial factors influence condom dispositions and joint condom use.

**Methods:**

We used data from a Dutch online sexual behaviour survey conducted in 2023 among 752 16–24 years old engaging in penile–vaginal sex. Partner type (one-time or recurring), condom use and psychosocial factors (attitudes and social norms regarding condom use and self-efficacy regarding condom use and condom negotiation) were assessed. Existing probabilistic maximum likelihood models were used and extended to estimate condom dispositions. For one-time partners, individuals were allocated a binary condom disposition: 0 (non-condom) and 1 (pro-condom). For recurring partners, dispositions ranged between 0 and 1. We estimated individual disposition distributions and condom use probabilities when people with opposite dispositions meet. Sex and psychosocial factors were incorporated to determine their influence on dispositions and condom use probabilities.

**Results:**

For both partner types, the model including attitudes fit the data best. For one-time partners, 96% of participants with strongly positive attitudes had pro-condom dispositions, compared with 54% of participants with moderately positive attitudes. The probability of using a condom when two opposite dispositions meet was 6%. For recurring partners, people with moderately positive attitudes showed only non-condom dispositions. In contrast, individuals with strongly positive attitudes exhibited more diverse dispositions, though they were mostly concentrated around pro-condom dispositions.

**Conclusions:**

Attitudes mostly influenced condom dispositions. One-time partnerships between persons with opposite dispositions rarely involve condom use. Interventions focusing on joint condom use decision-making and on improving attitudes towards condom use, particularly within one-time partnerships, may positively impact condom use among youth.

**WHAT IS ALREADY KNOWN ON THIS TOPIC**

- Research suggests that partners’ psychosocial factors can influence condom use, but many studies rely on individual perceptions of the partner’s characteristics. Limited research exists on how characteristics of different partner types, especially one-time partners, influence condom use. Innovative methods, such as statistical modelling can address these gaps and explore partner influences more comprehensively.

**WHAT THIS STUDY ADDS**

- The statistical approach estimated that attitudes best predicted condom use dispositions across partner types.
- Despite the majority of individuals having a pro-condom disposition with one-time partners, if individuals with opposite dispositions meet, condom use is highly unlikely.

**HOW THIS STUDY MIGHT AFFECT RESEARCH, PRACTICE OR POLICY**

- Our findings provide empirical condom use probabilities by partner disposition, enabling more realistic parameterisation of sexually transmitted infection (STI) transmission models.
- Our findings highlight the need for STI prevention strategies focused on improving attitudes towards condom use and strengthening joint condom use decision-making especially in one-time partnerships.

## Introduction

 Decreasing trends in condom use are observed among young people in many countries.^1–5^ A recent WHO report highlights these declining condom use rates as a cause of concern.^6^ To illustrate, while in 2012 74% of Dutch girls and 85% of boys reported using a condom during their most recent one-night stand, this decreased to 53% and 57%, respectively, in 2022.^7,8^ High population prevalences of chlamydia among youth^9–11^ and recent rises in gonorrhoea diagnoses^12^ emphasise the urgency for condom use interventions among youth.

Understanding and promoting condom use is challenging due to its dyadic nature and involvement of both partners’ influences. While most research focuses on how individual psychosocial factors influence condom use, few studies have assessed the role of a partner’s psychosocial factors. Available research suggests that a partner’s psychosocial factors can influence condom use decision-making,^13–15^ but these studies often rely on an individual’s perception of their partner rather than the partner’s actual perspective. This may not fully capture how both individuals influence joint condom use. Furthermore, studies on partner influences in new or one-time partnerships are scarce, possibly because participants cannot easily involve such partners in a study or may lack knowledge of their views. Partner type can influence both the frequency and nature of condom negotiation,^16,17^ consequently affecting condom use decision-making. The variability of how partner types may influence condom use, the challenges of studying new or one-time partners, and the cumbersome and time-consuming nature of partner studies highlight a clear need for alternative research methods to explore how partners influence condom use.

Hansson *et al*^18,19^ developed statistical methods to explore how partner factors influence condom use, by estimating individuals’ behavioural condom dispositions from sexual behaviour data. These condom dispositions are underlying tendencies towards condom use, and the combination of two individuals’ dispositions results in a joint condom use probability. The study of Hansson *et al*^18^ included sex to determine its influence on condom use dispositions, but found no differences in dispositions between females and males. However, other factors, such as psychosocial factors, were not taken into account.^18^ In this study, we extend the methods of Hansson *et al*^18,19^ by incorporating psychosocial factors, aiming to improve our understanding of how these influence condom use dispositions and joint condom use in various partner types.

## Methods

### Participants and recruitment

We used data from a sexual behaviour survey conducted in the Netherlands from April to June 2023. Details can be found elsewhere,^15^ and the survey can be found in online supplemental text S2. Participants were recruited through social media advertisement and were eligible if they were 16–24 years old and identified as heterosexual or bisexual, or if they engaged in vaginal sex with someone with the opposite anatomy. To limit recall bias, we only included participants who had vaginal sex in the past 12 months. Sex was determined based on participants’ sex assigned at birth (female or male), and no participants reported having undergone gender-affirming surgery.

### Patient and public involvement statement

Neither patients nor the public were involved in the design, conduct or dissemination of this study.

## Measures

### Demographics

Demographic characteristics assessed included age, sex assigned at birth, gender identity and sexual orientation. Gender identity and sexual orientation were collected to determine eligibility. Consistent with our aim to include sexually and gender-diverse youth, and acknowledging that penile–vaginal sex is not limited to cisgender heterosexual individuals, participants were included if they reported penile–vaginal sex within the past 12 months.

Educational level was classified according to the highest level completed within the Dutch educational system, grouped as lower, middle or higher. The lower category included primary education, prevocational secondary education, secondary vocational education level 1 and the first 3 years of senior general secondary or preuniversity education. The middle category included secondary vocational education levels 2, 3 or 4, and the final years of senior general secondary or preuniversity education. The higher category comprised higher vocational education and university.

Migration background was assessed based on the country or region of birth of the participant and both parents and, in accordance with Statistics Netherlands (CBS), classified into *no migration background*, *migrant* or *child of migrant*.

### Sexual partners, sex acts and condom use

Participants reported their number of vaginal sex partners during the past 12 months. For up to five partners, participants reported whether it was a one-time or recurring partner. For recurring partners, participants reported the duration of the partnership. Participants reported their sex frequency per week (duration <one month) or per month (duration≥one month). Condom use with a recurring partner was reported as *never/usually not/sometimes/usually/always;* with one-time partners condom use was reported as *yes/no/I don’t know*.

We calculated the total number of sex acts with a recurring partner as the number of sex acts per month (for partnerships lasting at least 1 month) multiplied by the partnership duration in months, or the number of sex acts per week (for partnerships lasting less than a month) multiplied by the partnership duration in weeks.

Condom use with a recurring partner was recoded into 0 (*never*), 0.25 (*usually not*), 0.5 (*sometimes*), 0.75 (*usually*) or 1 (*always*). For each recurring partnership, the number of condom protected sex acts was estimated by multiplying the calculated number of sex acts during the partnership by the numerical estimate of condom use.

### Psychosocial factors

Guided by the Theory of Planned Behavior^20^ and our previous research,^15^ the survey included assessments of attitudes, social norms and self-efficacy regarding condom use, and self-efficacy regarding condom use negotiation of the participant.^21^ These psychosocial constructs have been extensively examined in relation to condom use behaviour in previous research.^22–25^ The specific survey items were developed for the present study following the measurement recommendations of Conner and Norman.^26^ To reduce model complexity and improve usability and interpretability, psychosocial factors were dichotomised using a median split. We planned to label categories as negative versus positive or low versus high. However, examination of the distributions revealed that all constructs were skewed toward favourable values: attitudes and social norms were strongly skewed toward high/positive responses, and self-efficacy and negotiation self-efficacy were also skewed toward higher values. Accordingly, labels were applied as follows: *moderately positive* and *strongly positive* for attitudes and social norms, and *lower* and *higher* for self-efficacy and negotiation self-efficacy. In all cases, the lower category represents the lower half of a positively skewed distribution and should be interpreted as comparatively less favourable rather than neutral or negative.

Attitude towards condom use was measured with five semantic differential items, with responses indicated on 5-point bipolar response scales: What do you think about using condoms? Bad (1)—good (5), unpleasant (1)—pleasant (5), unenjoyable (1)—enjoyable (5), harmful (1)—beneficial (5) and foolish (1)—wise (5). Scores across the five items were summed and dichotomised into moderately positive and strongly positive.

We assessed injunctive (ie, others’ perceived approval) and descriptive (ie, others’ perceived behaviour) social norms with one item each: ‘People who are important to me think I should use a condom when I have sex’ (injunctive) and ‘People who are important to me use a condom when they have sex’ (descriptive). Responses were given on five-point scales (1=completely disagree, 5=completely agree). The scores from the two items were summed and dichotomised into moderately positive and strongly positive.

Condom use self-efficacy and condom use negation self-efficacy were each measured with one item: ‘I am confident that I can use a condom’ and ‘I am confident that I can convince a partner to use a condom’, respectively. Responses were given on a 5-point scale (1=completely disagree, 5=completely agree). Both measures were dichotomised into lower and higher.

### Statistical models

We used probabilistic models and a maximum likelihood approach based on Hansson *et al*,^18,19^ as described in online supplemental text S1. The models were extended to include psychosocial factors.

The primary outcome was the distribution of condom use dispositions, stratified by partner type (one-time or recurring). Condom dispositions are defined as underlying tendencies towards condom use or non-use, and thus can be observed as a behavioural pattern. These dispositions are not reported by the participant, but rather inferred from condom use data using statistical methods. We assumed that each individual had a condom disposition and that individuals’ dispositions were independent of each other. We also assumed that dispositions remain constant across all partners of the same type. For one-time partners, based on Hansson *et al*,^18,19^ we compared pro-con and pro-con-neutral models. The pro-con model assigned individuals to either a pro-condom or non-condom disposition, while the pro-con-neutral model included a neutral disposition. Model fit was assessed using log-likelihood and Akaike information criterion (AIC) values, where higher log-likelihood and lower AIC values indicate better fit. The model providing the best fit was considered the base model for one-time partners. For recurring partners, a geometric mean model was employed, with dispositions ranging between 0 (non-condom) and 1 (pro-condom). This model provided a beta distribution of disposition.

A secondary outcome was the probability of using a condom between two individuals with opposite dispositions (one-time partners only). We assumed that the interaction between two individuals’ dispositions determined condom use. For one-time partners with the same disposition, the probabilities were set as 0 (both non-condom), 0.5 (both neutral) or 1 (both pro-condom). Individuals with a neutral disposition either followed their partner’s disposition or had a 0.5 probability of condom use with a neutral disposition partner. For differing dispositions, the model estimated the condom use probabilities. For recurring partners, the probability of condom use in one act is given by the geometric mean of the two individuals’ dispositions. For recurring partners with the same disposition, their condom use probability was equal to their disposition value (eg, two individuals with disposition *x* had a combined probability of *x*). For differing dispositions, the geometric mean determined the probability (eg, dispositions of 0.2 and 0.7 yield a condom probability of $\sqrt{0.7\text{*}0.2}$ ≈ 0.37).

We first incorporated sex in the (base) model estimating the condom disposition distributions. Subsequently, we allowed condom probabilities to depend on sex, which could reveal whether one sex had a stronger influence on condom use probabilities. Next, the four dichotomous psychosocial factors were included separately to estimate condom use dispositions. We then allowed the condom use probability to depend on the psychosocial factors, which could determine individuals with the same disposition influenced the condom use probabilities differently depending on their psychosocial factors. The AIC values determined which models provided the best fit. CIs were not provided for any of the estimations due to model constraints and unequal participant data contributions. Instead, reliability was previously verified through a simulation study by Hansson *et al.*^18^

## Results

### Participant characteristics

In total, 1070 participants completed the survey, of whom 752 (70.3%) reported having had penile–vaginal sex in the past 12 months. Due to missing data caused by a survey routing error, the analytical sample consisted of 558 participants, of whom 494 (88.5%) had at least one recurring partner and 197 (35.3%) had at least one one-time partner. Participants were required to answer all questions, thus within the analytical sample, no data are missing. As individuals could report multiple partnerships, in total, data of 338 one-time partnerships and 645 recurring partnerships are included. Participant characteristics are presented in table 1.

**Table 1:** Demographic and sexual behaviour characteristics, and psychosocial factors of Dutch youth who had recent sex, recruited through an online sexual behaviour survey in 2023

	Assigned female at birth (n=327)	Assigned male at birth (n=231)	Total (n=558)
Age (mean, SD)	19.0 (2.1)	18.7 (1.7)	18.9 (2.0)
Level of highest education completed			
Low	102 (31%)	68 (29%)	170 (30%)
Middle	174 (53%)	139 (60%)	313 (56%)
High	51 (16%)	24 (10%)	75 (13%)
Migration background			
No migration background	246 (75%)	180 (78%)	426 (76%)
Migrant	23 (7%)	14 (6%)	37 (7%)
Child of migrant(s)	58 (18%)	37 (16%)	95 (17%)
Number of one-time partners past 12 months			
0	221 (68%)	140 (61%)	361 (65%)
1	61 (19%)	49 (21%)	110 (20%)
2	20 (6%)	27 (12%)	47 (8%)
3	20 (6%)	6 (3%)	26 (5%)
4	5 (2%)	9 (4%)	14 (3%)
Number of recurring partners past 12 months			
0	30 (9%)	34 (15%)	64 (11%)
1	224 (69%)	162 (70%)	386 (69%)
2	52 (16%)	22 (10%)	74 (13%)
3	15 (5%)	10 (4%)	25 (4%)
4	6 (2%)	3 (1%)	9 (2%)
Proportion of condom protected sex acts with recurring partners, mean, SD	0.46 (0.40)	0.41 (0.39)	0.44 (0.40)
Proportion of condom protected sex acts with one-time partners, mean, SD	0.50 (0.46)	0.52 (0.46)	0.51 (0.44)
Attitudes towards condom use			
Moderately positive	163 (50%)	156 (68%)	319 (57%)
Strongly positive	164 (50%)	75 (32%)	239 (43%)
Condom use social norms			
Moderately positive	171 (54%)	139 (60%)	310 (56%)
Strongly positive	156 (48%)	92 (40%)	248 (44%)
Condom use self-efficacy			
Lower	196 (60%)	102 (44%)	297 (53%)
Higher	132 (40%)	129 (56%)	261 (47%)
Condom use negotiation self-efficacy			
Lower	171 (52%)	139 (60%)	314 (56%)
Higher	156 (48%)	92 (40%)	244 (44%)

### One-time partners

For one-time partners, the pro-con model provided a better fit than the pro-con-neutral model and was therefore selected as base model (online supplemental table 1). We found that 70% of participants had a pro-condom disposition, with a 3% probability of condom use between partners with opposite dispositions (table 2). Adding sex to the model did not improve fit, indicating that condom dispositions are not influenced by sex and sex was not further incorporated (online supplemental table 1).

**Table 2:** Condom dispositions and probabilities in one-time partners

	LogL	AIC	Proportion of participants with a pro-condom disposition	Probability of using a condom when opposite dispositions meet
Pro-con model	−185.0	374.0	0.70	0.03
Pro-con model including sex	−184.6	377.3		0.02
Females			0.72	
Males			0.68	
Model including attitudes only	−173.5	353.0		0.06
Moderately positive			0.54	
Strongly positive			0.96	
Model including norms only	−181.8	369.6		0.03
Moderately positive			0.60	
Strongly positive			0.82	
Model including self-efficacy only	−184.5	375.0		0.01
Lower			0.65	
Higher			0.74	
Model including negotiation self-efficacy only	−183.2	372.4		0.01
Lower			0.63	
Higher			0.79	

AIC, Akaike information criterion.

When looking at the models incorporating psychosocial factors (table 2), the model including attitudes fit the data best. Among participants with moderately positive attitudes 54% had a pro-condom disposition, compared with 96% among participants with strongly positive attitudes (table 2). The estimated condom use probability between two individuals with opposite dispositions was 6%. The models that included norms, self-efficacy or negotiation self-efficacy also fit better than the base model, but less than the attitudes model. Higher proportions of pro-condom dispositions were found in individuals with strongly positive norms, or higher self-efficacy or negotiation self-efficacy. Condom use probabilities between individuals with opposite dispositions were also low.

We allowed the condom use probability to depend on the four different psychosocial factors (online supplemental table S1). This would establish if individuals with, for example, strongly positive attitudes had greater influence on condom use than those with negative attitudes. As this decreased model fit, we determined the psychosocial factors only affected the distribution of condom dispositions but not the condom use probabilities.

### Recurring partners

The base model for recurring partners was highly skewed towards 0 (non-condom dispositions), with a beta distribution of α=0.36 and β=0.72 and a mean disposition of 0.33 (table 3, figure 1A), indicating that the majority of individuals had non-condom dispositions with recurring partners.

**Figure 1:**
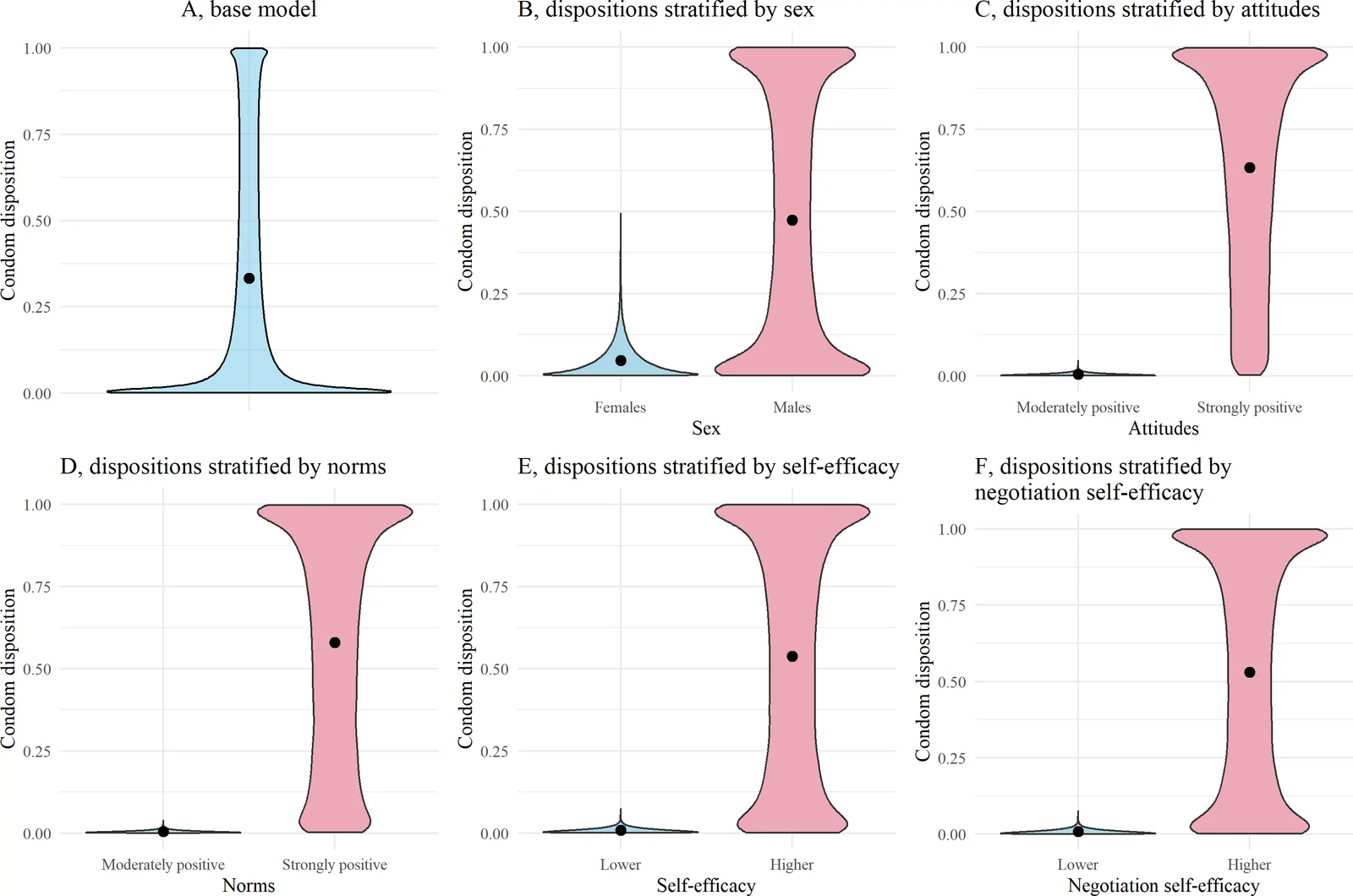
Density plots for the distributions of condom dispositions in recurring partners. The x-axis reflects the density, the y-axis reflects the condom disposition values and the dot reflects the median.

**Table 3:** Beta distributions of condom dispositions in recurring partners ranging between 0 (complete non-condom disposition) and 1 (complete pro-condom disposition)

	LogL	AIC	α	β	Mean condom disposition
Base model	−949.20	1902.40	0.36	0.72	0.33
Model including sex	−927.96	1863.93			
Females			0.75	15.24	0.05
Males			0.38	0.43	0.47
Model including attitudes only	−826.28	1660.56			
Moderately positive			1.30	279.48	0.01
Strongly positive			0.94	0.55	0.63
Model including attitudes and sex	−834.10	1684.21			
Moderately positive attitudes, female			1.39	308.16	0.00
Strongly positive attitudes, female			0.81	0.55	0.60
Moderately positive attitudes, male			1.41	319.64	0.00
Strongly positive attitudes, male			1.00	0.56	0.64
Model including norms only	−865.23	1738.46			
Moderately positive			1.40	314.03	0.00
Strongly positive			0.67	0.48	0.58
Model including norms and sex	−885.71	1787.42			
Moderately positive norms, female			0.68	10.40	0.06
Strongly positive norms, female			0.51	0.47	0.52
Moderately positive norms, male			1.05	74.53	0.01
Strongly positive norms, male			0.70	0.44	0.61
Model including self-efficacy only	−896.92	1801.85			
Lower self-efficacy			1.42	161.48	0.01
Higher self-efficacy			0.54	0.46	0.54
Model including self-efficacy and sex	−922.18	1860.37			
Lower self-efficacy, female			0.35	0.55	0.39
Higher self-efficacy, female			0.55	1.48	0.27
Lower self-efficacy, male			1.04	67.13	0.02
Higher self-efficacy, male			0.40	0.50	0.44
Model including negotiation self-efficacy only	−893.65	1795.30			
Lower negotiation self-efficacy			1.40	156.06	0.01
Higher negotiation self-efficacy			0.50	0.44	0.53
Model including negotiation self-efficacy and sex	−896.87	1809.74			
Lower negotiation self-efficacy, female			0.35	0.53	0.40
Higher negotiation self-efficacy, female			0.39	0.36	0.52
Lower negotiation self-efficacy, male			1.16	139.86	0.01
Higher negotiation self-efficacy, male			0.57	2.35	0.20

AIC, Akaike information criterion.

Incorporating sex improved the fit (table 3). While females rarely had pro-condom dispositions in recurring partnerships, males did (figure 1B). Next, we modelled the four separate psychosocial factors combined with sex, showing that these four models demonstrated better fit than the model including sex only (table 3). However, the four models including only the psychosocial factors fit better than the models that also included sex; the model including only attitudes demonstrating the best fit compared with the three other psychosocial factors (table 3). The distributions of the dispositions varied by psychosocial factors (table 3, figure 1C–F): dispositions of participants with moderately positive attitudes, moderately positive norms, lower self-efficacy or lower negotiation self-efficacy were skewed towards 0, not exceeding 0.1. Dispositions of participants with strongly positive attitudes or norms, higher self-efficacy or negotiation self-efficacy were skewed towards 1, but also showed values near 0.

## Discussion

We used statistical models to infer individuals’ behavioural condom dispositions and condom use probabilities when opposites met, taking sex and psychosocial factors into account. Most participants had a pro-condom disposition with one-time partners, while most had a non-condom disposition with recurring partners. The models including psychosocial factors outperformed the models including sex, and the model incorporating attitudes towards condom use gave the best fit for both partner types. Non-condom dispositions were mostly found in participants with moderately positive attitudes, while pro-condom dispositions were found in those with strongly positive attitudes. Joint condom use was low in one-time encounters between partners with opposing dispositions.

The key strength of this study is the addition of psychosocial factors to existing models. This improved the model fit and enabled a more comprehensive understanding of factors influencing condom dispositions. Additionally, we replicated previously developed statistical models using independent data and found that the models effectively estimated condom dispositions, thereby validating their robustness. Furthermore, our study included a demographically diverse sample, which enhanced the reliability and comprehensiveness of our findings. However, there are also some limitations. First, we could only include participants with at least one new partner in the past year, due to a survey routing error. However, we still had data on 144 partnerships lasting over 1 year. Since most Dutch youth stop using condoms after 3 months,^7^ we expect little influence of the underrepresentation of longer lasting partnerships on condom dispositions in recurring partners. Second, we limited the number of recent partners to five to reduce survey completion time and recall bias. However, only 8% reporting having had more partners and condom use with additional partners might not have been different from the reported five, we expect no influence on the results. Third, measures of condom use with each sexual partner were self-reported and may therefore be subject to social desirability bias. This bias may have affected reports of condom use differently for one-time versus recurring partners, as participants may have experienced greater normative pressure to report condom use with one-time partners. Although confidentiality was emphasised throughout the survey, social desirability bias cannot be fully eliminated. However, the fact that a substantial amount of participants reported non-condom use suggests that respondents felt able to disclose less socially desirable behaviours, which may indicate that this bias was not pervasive. Finally, no adjustments were made for potential confounding sociodemographic variables. However, this is in line with the Theory of Planned Behavior. Within this framework, sociodemographic characteristics are conceptualised as background factors that influence behaviour indirectly through psychosocial constructs such as attitudes, social norms and self-efficacy.^20^ Adjusting for these variables could therefore result in overadjustment and unnecessarily complicate the statistical models. Nevertheless, we acknowledge that sociodemographic and contextual factors (eg, condom availability, privacy concerns and familial influences) may affect condom use and inferred condom dispositions, particularly among youth.

Our results are consistent with previously published studies. Consistent with our findings for one-time partnerships, Hansson *et al*^18^ found no sex differences in condom dispositions among heterosexuals. However, our study showed slightly higher non-condom dispositions in one-time partnerships: 30% vs 25% in their study.^18^ This can be explained by the use of different data; Hansson *et al* used data of sexually transmitted infection (STI) clinic visitors aged 15–20, who are typically engaged in higher STI risk behaviour, while we used data of more general sexually active youth. Our study did find differences in condom dispositions with recurring partners between females and males, with females having more non-condom dispositions than males. This was unexpected, as females are known to, for example, take less risks than males.^27^ A possible explanation can be found in the use of hormonal contraception. In recurring partnerships, STI prevention may become less relevant, with females often relying more on hormonal contraception. However, since we did not have data on contraceptive use, this is hypothetical.

Our study highlights the low condom use probability (6% in the attitudes model) in one-time encounters between partners with opposing dispositions. This is lower than usually incorporated in STI transmission models estimating the impact of intervention measures on STI prevalence.^28–30^ The found condom use probability in mixed-disposition pairs can help refine model accuracy and improve projections. However, we emphasise that this low rate applies specifically to mixed-disposition pairs. Because most participants had a pro-condom disposition, overall condom use in one-time partnerships is higher. For example, based on the proportions of individuals with pro-condom and non-condom dispositions and the condom use probabilities, approximately 50% of one-time sexual partners involve a condom, consistent with reported behaviour.^7^

For one-time partners, psychosocial factors influenced the distribution of condom dispositions, but not how strongly an individual’s disposition affected joint condom use. This suggests that, for example, attitudes do affect dispositions, but when partners with opposite dispositions meet, the condom probability remains unaffected by their attitudes. To illustrate, an individual with a pro-condom disposition and strongly positive attitudes does not have more influence on joint condom use with a non-condom-disposition partner than an individual with a pro-condom disposition and moderately positive attitudes. This is especially interesting for negotiation self-efficacy, where one might expect greater influence from those confident in negotiating condom use. However, a discrepancy may exist between feeling capable and actually acting on it. In addition, self-efficacy, and presumably negotiation self-efficacy, are generally only relevant if someone already is motivated to use condoms.^31^

For recurring partners, we observed a large amount of non-condom dispositions, indicating that many participants tend to not use condoms with recurring partners. This aligns with previous literature indicating that Dutch youth are more likely to use condoms with one-night stands than with recurring partners.^7^ However, we also observed some pro-condom dispositions in recurring partnerships, possibly reflecting specific contexts. For example, condoms may still be used in the early stages of a partnership^7^ with a recurring but non-committed partner where the perceived risk for STI may be higher,^32^ or in longer partnerships where condoms are used as a method of contraception. This may explain why, despite most individuals having a non-condom disposition in recurring partnerships, some still have a pro-condom disposition.

For both partner types, including attitudes in the models provided the best fit, consistent with research identifying attitudes as the strongest predictor of condom use intentions, surpassing norms and self-efficacy.^25^ By linking attitudes directly to actual condom use, our study bypassed common discrepancies between intentions and behaviour.^33^ While the other psychosocial factors appear more context-dependent,^34^ attitudes appear relatively stable, which may explain their significant impact on condom dispositions across partner types.^34^ These findings highlight the importance of psychosocial factors, particularly attitudes, in condom use behaviour and suggest that they could serve as effective targets for interventions.

Given the central role of attitudes, youth-oriented condom promotion interventions should focus on fostering positive attitudes toward condom use. According to the Behaviour Change Wheel, this can be achieved through persuasion (eg, using credible sources or sharing information about health consequences) and enablement (eg, problem solving, action planning or self-monitoring).^35^ Possible interventions in formal settings, such as schools, could incorporate expert-led sessions or interactive workshops from health professionals. Games or tools using avatars could allow young people to practice condom negotiation in realistic scenarios, such as responding to a partner who is reluctant to use condoms. Outside formal settings, condom use can be promoted through social media campaigns highlighting its benefits or youth-friendly health services sharing relatable role model stories^36^ that resonate with young people and reinforce positive condom attitudes. These are examples of possible interventions, but it is important to tailor approaches to the specific context of the target population.

Our study emphasises the role of psychosocial factors, particularly attitudes, in condom dispositions during penile–vaginal sex across different partnership types, specifically among young individuals engaging in sexual activity with partners of opposite sex. We improved our understanding and estimation of condom use behaviours, emphasising the potential of attitudes as intervention targets. Despite the majority of participants having pro-condom dispositions with one-time partners, low condom probabilities were observed in encounters involving opposite dispositions, underscoring the need for interventions focusing on joint decision-making strategies in pairs with opposite preferences.

## Supplementary material

10.1136/bmjph-2025-003606 online supplemental file 1

## Data Availability

Data are available upon reasonable request. All data relevant to the study are included in the article or uploaded as supplementary information.
