# Supplementary material for: Influence of individual and partner preferences on joint condom use during vaginal sex among young people in the Netherlands: a statistical modelling study
Source: BMJ Public Health. 2026 Oct 7;4(4):e003606. doi: 10.1136/bmjph-2025-003606 (PMC13647015; doi:10.1136/bmjph-2025-003606)
Supplement: Supplementary file 1 [file bmjph-4-4-s001.docx]

**Supplementary Material**

**The influence of individual and partner preferences on joint condom use during vaginal sex among young people in the Netherlands: a statistical modelling study**

Alcira de Vries, MSc., Disa Hansson, PhD, Prof. Dr. John B.F. de Wit, Chantal den Daas ,PhD, Janneke C.M. Heijne, PhD

Contents

[S1. Description of the models	2](#_Toc223087837)

[S1.1 One-time partners	3](#_Toc223087838)

[S1.2. Recurring sex partners	11](#_Toc223087839)

[Table S1. Overview of all models estimating condom dispositions and probabilities in one-time partners.	14](#_Toc223087840)

[S2. Questionnaire, translated from Dutch	16](#_Toc223087841)

[S3. STROBE checklist	31](#_Toc223087842)

[References	35](#_Toc223087843)

# S1. Description of the models

The models used in the analysis is based on and extends previous work by Hansson et al. where disposition models for heterosexual [1] and same-sex data [2] were derived. The models are extended to incorporate psychosocial variables. Note that the models were designed to estimate the proportions of non-condom dispositions and the probabilities of not using a condom. In the manuscript we describe pro-condom dispositions and probabilities of using a condom to enhance interpretability.

In the models, it is assumed that each individual $i$ has a condom disposition $x_{i}$ (which distribution we aim to infer), and that condom use (*observation*) is determined by a combination of two individuals’ dispositions. An individual who always wants to use condoms has a disposition of 0 and an individual who never wants to use condom has a disposition 1. Not using condoms use is decided by the probability:

$$P\left( no condom | x_{i},x_{j} \right).$$

Hence, there are two parts of the modelling; first the distribution of the disposition and second how two individuals’ distributions generate the probability of condom use.

Assumptions: The disposition does not depend on the number of partners of individuals and the number of partners does not depend on the disposition. We further assume that the condom disposition for one-time partners is independent of the condom disposition in a recurring partner.

We will consider two different sets of models, one for one-time partners and one for recurring partners. For one-time partners, we used the reported number of one-time sex partners during the past year and whether a condom was used; and for recurring sex partners the we used the number of recurring partners, the number of sex acts per partner and in how many of the sex acts a condom was used. For each individual, we also used the dichotomized psychological variables (attitudes, norms, self-efficacy and negotiation self-efficacy)

## S1.1 One-time partners

We will in the following explain the model for one-time partners. For each individual $i=1,\ldots,n$ in the sample of size $n$, the data consists of the number of one-time sex partners and the number of one-time partners with whom a condom was used. Each individual $i$ in the sample also has a known attribute $A_{i}$ (e.g. attitudes) which can take two values ($A_{0}$ indicating less positive attitudes or $A_{1}$indicating more positive attitudes). The distribution of condom dispositions may be different depending on the attribute. Let $Z_{d}$ denote the number of non-condom contacts of an individual with $d$ one-time sex partners. The likelihood not using a condom will be given by the product of probabilities of observing each individual’s number of non-condom acts among its $d$ partners. If we denote individual $i$’s number of non-condom contacts by $k_{i}$ and its total number of one-time partners by $d_{i},$ the likelihood is given by

$$\prod_{i=1}^{n} P\left( Z_{d_{i}}=k_{i} \right|A_{i}).$$

An individual can be one of two types, pro-condom (for the use of condoms) or non-condom (against the use of condom). If two individuals with a pro-condom dispositions meet they will use a condom; if two individuals with a non-condom disposition meet they will not use a condom; and if two individuals with opposite dispositions meet, they will use a condom, i.e.:

$$\varepsilon=P(no condom when two individuals of opposite disposition meet)=0.$$

What the derived model aims to infer is the condom disposition distribution and in this setting the fraction of individuals that have a non-condom disposition, denoted $p$, and the fraction of individuals with a pro-condom disposition, $1-p.$ We will also let $\varepsilon$ be a free parameter to infer.

The probability that for individual $i$ in the sample observe $k$ non-condom contacts among $d$ contacts, is

$$P(Z_{d}=k)= P\left( Z_{d}=k | x_{i}=0 \right)(1-p)+P\left( Z_{d}=k | x_{i}=1 \right)p.$$

We begin with the probability to observe $k$ non-condom contacts among $d$ contacts in total given that individual $i$ has a pro-condom disposition (disposition $x_{i}=0$). We need to condition on the type of the $d$ partners. However, we have some extra information. When the individual has a pro-condom disposition, we can only observe non-condom contacts when the partner $j$ has a non-condom disposition ($x_{j}=1$). Hence, individual $i$ can have met at *most* $d-k$ other individuals with a pro-condom disposition and at *least* $k$ individuals with a non-condom disposition. We condition on how many $m\in\{0,\ldots,d-k\}$ partners with a pro-condom disposition that individual $i$ had. Among the $d-m$ partners with a non-condom disposition individual $i$ met, a condom should not have been used with $k$ of them ($\varepsilon^{k}$) and a condom should have been used with $d-k-m$ of them ($\varepsilon^{d-k-m}$).

$$P\left( Z_{d}=k | x_{i}=0 \right)= \sum_{m=0}^{d-k} \left( \begin{matrix} d \\ m \end{matrix} \right){\left( \begin{matrix} d-m \\ d-m-k \end{matrix} \right)\left( 1-p \right)}^{m}p^{d-m}\varepsilon^{k}{(1-\varepsilon)}^{d-k-m}. (1)$$

Next, given that individual $i$ has a non-condom disposition ($x_{i}=1$), the individual cannot have met more than $k$ partners with a non-condom disposition for us to observe $k$ non-condom contacts. Therefore, condition on how many $m\in\{0,\ldots,k\}$ of the $d$ partners have a non-condom disposition. With all $m$ partners with a non-condom disposition a condom will not be used, and with the remaining $d-m$ partners with a pro-condom disposition $k-m$ needs to be without condom in order for us to observe $k$ non-condom contacts:

$$P\left( Z_{d}=k | x_{i}=1 \right)= \sum_{m=0}^{k} \left( \begin{matrix} d \\ m \end{matrix} \right)\left( \begin{matrix} d-m \\ k-m \end{matrix} \right)p^{m}{(1-p)}^{d-m}\varepsilon^{k-m}{(1-\varepsilon)}^{d-k}. (2)$$

To be clear regarding $k=0:$

$$P\left( Z_{d}=0 | x_{i}=0 \right)=\sum_{m=0}^{d} \left( \begin{matrix} d \\ m \end{matrix} \right)\left( 1-p \right)^{m}p^{d-m}{(1-\varepsilon)}^{d-m},$$

$$P\left( Z_{d}=0 | x_{i}=1 \right)= {(1-p)}^{d}{(1-\varepsilon)}^{d} .$$

*Incorporating sex*
To account for sex, we allow for females and males to draw their condom dispositions from different distributions, i.e. the fraction of individuals with a pro-condom disposition may be different for females and males. The dispositions of males and females are assumed independent. We additionally create models that let $\varepsilon$ depend on if it is the male or female that is for or against by including $\varepsilon_{NC}^{FM}$ and $\varepsilon_{NC}^{MF}$where

$$\varepsilon_{NC}^{WF}=P\left( no condom | the female has a non-condom disposition and the male has a pro-condom disposition \right),$$

$$\varepsilon_{NC}^{MF}=P\left( no condom | the male has a non-condom disposition and the female a pro-condom disposition \right).$$

More details can be found elsewhere ^[1]^

*Incorporating psychosocial variables*

We will refer to the psychosocial factor as *attribute*. We assume that the attribute $A$ can take two values ($A_{0}$ or $A_{1}$), we then allow for the condom disposition distribution to be different depending on if $A=A_{0}$or $A=A_{1}$. We also allow for that the non-condom probability given that two individuals of opposite disposition meet may depend on the attribute variable *A* as well:

$$\varepsilon_{NC}^{A_{0}A_{0}}=P\left( no condom | the individual with a non-condom disposition is A_{0} and the individual with a pro-condom disposition is A_{0} \right),$$

$$\varepsilon_{NC}^{A_{1}A_{0}}=P\left( no condom | the individual with a non-condom disposition is A_{1} andthe individual with a pro-condom disposition is A_{0} \right),$$

$$\varepsilon_{NC}^{A_{0}A_{1}}=P\left( no condom | the individual with a non-condom disposition A_{0} and the individual with a pro-condom disposition is A_{1} \right),$$

$$\varepsilon_{NC}^{A_{1}A_{1}}=P\left( no condom | the individual with a non-condom disposition is A_{1} and the individual with a pro-condom disposition is A_{1} \right).$$

Denote the probability that an individual (proportion of individuals that) has attribute $A_{0}$ and $A_{1}$ are given by $q_{A_{0}}$and $q_{A_{1}}$respectively (so that $q_{A_{0}}+ q_{A_{1}}=1$). Let us further denote the probability that an individual with attribute $A_{0}$ has a non-condom disposition by $p_{A_{0}}$and similarly the probability that an individual with attribute $A_{1}$ is has a non-condom disposition by $p_{A_{1}}$, that is

$$p_{A_{0}}:=P\left( individual with a non˗condom disposition | {A=A}_{0} \right),$$

$$p_{A_{1}}:=P\left( individual with a non˗condom disposition| {A=A}_{1} \right).$$

Then we have that the fraction individuals with a pro-condom disposition with attribute $A_{0}$ is equal to $q_{A_{0}}-p_{A_{0}}$ and the fraction individuals with a pro-condom disposition with attribute $A_{1}$ is equal to $q_{A_{1}}-p_{A_{1}}$.

Remember that an individual with a pro-condom disposition is given by disposition $x=0$, and a non-condom disposition is given disposition $x=1.$The probability that we for an individual with attribute $A=A_{y}$ ($y\in\{0,1\}$) observe $k$ non-condom contacts among $d$ contacts is

$P\left( Z_{d}=k | A=A_{y} \right)= P\left( Z_{d}=k | A=A_{y}, x=0 \right)\left( 1-p_{A_{y}} \right)+P\left( Z_{d}=k | A=A_{y}, x=1 \right)p_{A_{y}}.$ (3)

The two main probabilities here, $P\left( Z_{d}=k | A=A_{y}, x=0 \right)$ and $P\left( Z_{d}=k | A=A_{y}, x=1 \right)$, will be similar to Equation (1) and Equation (2), where we needed to condition on the disposition type of the partners, but we do now further need to condition on the attribute of the partners. Since the fraction of individuals with a non-condom disposition are different depending on attribute, and that the non-condom probability when two individuals with opposite dispositions meet depend on their attributes, we need to condition on the attribute among the condom outcomes and the non-condom outcomes.

***The case when individual*** $\boldsymbol{i}$***, with attribute***$\boldsymbol{A}_{\boldsymbol{y}}$ ***has a non-condom disposition***

In Equation (2), we look at an individual $i$ with a non-condom disposition and condition on that this individual met $m$ partners with a non-condom disposition and therefore $d-m$ partners with a pro-condom disposition. Now we further need to condition on the attribute of the partners to find $P\left( Z_{d}=k | A=A_{y}, x=1 \right)$.

We begin with the $m$ partners with a non-condom disposition, an individual with a non-condom disposition who meets another individual with a non-condom disposition will not use condom and there will therefore be $m$ non-condom contacts among these $m$ partners, hence we only need to condition on the attribute of these $m$partners with a non-condom disposition. If we condition on that $l_{n}\in\{0,\ldots,m\}$ of the $m$ partners with a non-condom disposition have attribute $A_{0}$ (and the remaining $m-l_{n}$ are $A_{1}$), then the term $p^{m}$ in Equation (2) will be changed to

$$\sum_{l_{n}=0}^{m} \left( \begin{matrix} m \\ l_{n} \end{matrix} \right)q_{A_{0}}^{l_{n}}q_{A_{1}}^{m-l_{n}}p_{A_{0}}^{l_{n}} p_{A_{1}}^{m-l_{n}}.$$

Now, for the $d-m$ partners with a pro-condom disposition, we will need to change the term

$${(1-p)}^{d-m}\varepsilon^{k-m}{(1-\varepsilon)}^{d-k}=\left( \left( 1-p \right)\varepsilon\right)^{k-m}\left( \left( 1-p \right)(1-\varepsilon) \right)^{d-k}$$

from Equation (2). With the same arguments as for the model disregarding attributes, in Equation (2), individual $i$ can not use condom with $k-m$ of the partners with a pro-condom disposition and must use condom with $d-m-\left( k-m \right)=d-k$ of the partners with a pro-condom disposition. To take attributes into account, Equation (2) needs to be changed by conditioning on how many of the partners with a pro-condom disposition that individual $i$ used condom with had attribute $A_{0}$ and $A_{1}$, and we need to condition on how many of the partners with a pro-condom disposition that individual $i$ did not use a condom with had attribute $A_{0}$ and $A_{1}$. Conditioning on that $l_{c}\in\{0,\ldots,k-m\}$ of the $k-m$ condom contacts have attribute $A_{0}$, then the term $\left( \left( 1-p \right)\varepsilon\right)^{k-m}$ in Equation (2) will change to

$$\sum_{l_{c}=0}^{k-m} \left( \begin{matrix} k-m \\ l_{c} \end{matrix} \right)q_{A_{0}}^{l_{c}}q_{A_{1}}^{k-m-l_{c}}\left( 1-p_{A_{0}} \right)^{l_{c}}\left( 1-p_{A_{1}} \right)^{{k-m-l}_{c}}\left( \varepsilon_{NC}^{A_{y}A_{0}} \right)^{l_{c}}\left( \varepsilon_{NC}^{A_{y}A_{1}} \right)^{{k-m-l}_{c}}.$$

Similarly, we need to condition on how many of the $l_{c}\in\{0,\ldots,d-k\}$ of the $d-k$ condom contacts (among the partners with a pro-condom disposition) that have attribute $A_{0}$, then the term $\left( \left( 1-p \right)(1-\varepsilon) \right)^{d-k}$ in Equation (2) will be changed to

$$\sum_{l_{c}=0}^{d-k} \left( \begin{matrix} d-k \\ l_{c} \end{matrix} \right)q_{A_{0}}^{l_{c}}q_{A_{1}}^{d-k-l_{c}}\left( 1-p_{A_{0}} \right)^{l_{c}}\left( 1-p_{A_{1}} \right)^{{d-k-l}_{c}}\left( 1-\varepsilon_{NC}^{A_{y}A_{0}} \right)^{l_{c}}\left( 1-\varepsilon_{NC}^{A_{y}A_{1}} \right)^{{d-k-l}_{c}}.$$

Combining it all gives us:

$$P\left( Z_{d}=k | A=A_{y}, x=1 \right)=$$

$$\sum_{m=0}^{k} \left( \begin{matrix} d \\ m \end{matrix} \right)\left( \begin{matrix} d-m \\ k-m \end{matrix} \right)\left( \sum_{l_{n}=0}^{m} \left( \begin{matrix} m \\ l_{n} \end{matrix} \right)q_{A_{0}}^{l_{n}}q_{A_{1}}^{m-l_{n}}p_{A_{0}}^{l_{n}} p_{A_{1}}^{m-l_{n}} \right)\times$$

$$\times\left( \sum_{l_{c}=0}^{k-m} \left( \begin{matrix} k-m \\ l_{c} \end{matrix} \right)q_{A_{0}}^{l_{c}}q_{A_{1}}^{k-m-l_{c}}\left( 1-p_{A_{0}} \right)^{l_{c}}\left( 1-p_{A_{1}} \right)^{{k-m-l}_{c}}\left( \varepsilon_{NC}^{A_{y}A_{0}} \right)^{l_{c}}\left( \varepsilon_{NC}^{A_{y}A_{1}} \right)^{{k-m-l}_{c}} \right)\times$$

$$\times\left( \sum_{l_{c}=0}^{k-m} \left( \begin{matrix} k-m \\ l_{c} \end{matrix} \right)q_{A_{0}}^{l_{c}}q_{A_{1}}^{k-m-l_{c}}\left( 1-p_{A_{0}} \right)^{l_{c}}\left( 1-p_{A_{1}} \right)^{{k-m-l}_{c}}\left( 1-\varepsilon_{NC}^{A_{y}A_{0}} \right)^{l_{c}}\left( 1-\varepsilon_{NC}^{A_{y}A_{1}} \right)^{{d-k-l}_{c}} \right).$$

***The case when individual*** $\boldsymbol{i}$***, with attribute***$\boldsymbol{A}_{\boldsymbol{y}}$ ***is a condom individual***

In Equation (1), we look at a condom individual $i$ and condition on that this individual met $m$ partners with a pro-condom disposition and $d-m$ partners with a non-condom disposition. Now we further need to condition on the attribute of the partners to find $P\left( Z_{d}=k | A=A_{y}, x=0 \right)$. To observe $k$ non-condom contacts, as in Equation (1), among the $d-m$ partners with a non-condom disposition individual $i$ met, a condom should not have been used with $k$ of them and a condom should have been used with $d-k-m$ of them.

We begin with the $m$ partners with a pro-condom disposition, a condom individual who meets another individual with a pro-condom disposition will use a condom, and there will therefore be $m$ condom contacts among these $m$ partners. We therefore only need to condition on the attribute of these $m$ partners with a pro-condom disposition. If we condition on that $l_{c}\in\{0,\ldots,m\}$ of the $m$ condom partners have attribute $A_{0}$ (and the remaining $m-l_{c}$ are $A_{1}$), then the term $\left( 1-p \right)^{m}$ in Equation (1) will be changed to

$$\sum_{l_{c}=0}^{m} \left( \begin{matrix} m \\ l_{c} \end{matrix} \right)q_{A_{0}}^{l_{c}}q_{A_{1}}^{m-l_{c}}\left( 1-p_{A_{0}} \right)^{l_{c}}\left( 1-p_{A_{1}} \right)^{m-l_{c}}.$$

Next, we look at the $d-m$ non-condom partners. Here we will change the term

$$p^{d-m}\varepsilon^{k}{(1-\varepsilon)}^{d-k-m}= {(p\varepsilon)}^{k}({p(1-\varepsilon))}^{d-k-m}$$

from Equation (1). Conditioning on that $l_{n}\in\{0,\ldots,k\}$ of the partners with a non-condom disposition have attribute $A_{0}$, changes the term ${(p\varepsilon)}^{k}$ from Equation (1) to

$$\sum_{l_{n}=0}^{k} \left( \begin{matrix} k \\ l_{n} \end{matrix} \right)q_{A_{0}}^{l_{n}}q_{A_{1}}^{k-l_{n}} p_{A_{0}}^{l_{n}}p_{A_{1}}^{{k-l}_{n}}\left( \varepsilon_{NC}^{A_{0}A_{y}} \right)^{l_{n}}\left( \varepsilon_{NC}^{A_{1}A_{y}} \right)^{{k-l}_{n}}.$$

Conditioning on that $l_{n}\in\{0,\ldots,d-k-m\}$ of the $d-k-m$ partners with a non-condom disposition have attribute $A_{0}$, changes the term $({p(1-\varepsilon))}^{d-k-m}$ from Equation (1) to

$$\sum_{l_{n}=0}^{d-k-m} \left( \begin{matrix} d-k-m \\ l_{n} \end{matrix} \right)q_{A_{0}}^{l_{n}}q_{A_{1}}^{d-k-m-l_{n}} p_{A_{0}}^{l_{n}}p_{A_{1}}^{{d-k-m-l}_{n}}\left( {1- \varepsilon}_{NC}^{A_{0}A_{y}} \right)^{l_{n}}\left( 1-\varepsilon_{NC}^{A_{1}A_{y}} \right)^{{d-k-m-l}_{n}}.$$

This all together yields that

$$P\left( Z_{d}=k | A=A_{y}, x=0 \right)=$$

$$\sum_{m=0}^{d-k} \left( \begin{matrix} d \\ m \end{matrix} \right)\left( \begin{matrix} d-m \\ k-m-k \end{matrix} \right)\left( \sum_{l_{c}=0}^{m} \left( \begin{matrix} m \\ l_{c} \end{matrix} \right)q_{A_{0}}^{l_{c}}q_{A_{1}}^{m-l_{c}}\left( 1-p_{A_{0}} \right)^{l_{c}}\left( 1-p_{A_{1}} \right)^{m-l_{c}} \right)\times$$

$$\times\left( \sum_{l_{n}=0}^{k} \left( \begin{matrix} k \\ l_{n} \end{matrix} \right)q_{A_{0}}^{l_{n}}q_{A_{1}}^{k-l_{n}} p_{A_{0}}^{l_{n}}p_{A_{1}}^{{k-l}_{n}}\left( \varepsilon_{NC}^{A_{0}A_{y}} \right)^{l_{n}}\left( \varepsilon_{NC}^{A_{1}A_{y}} \right)^{{k-l}_{n}} \right)\times$$

$$\times\left( \sum_{l_{n}=0}^{d-k-m} \left( \begin{matrix} d-k-m \\ l_{n} \end{matrix} \right)q_{A_{0}}^{l_{n}}q_{A_{1}}^{d-k-m-l_{n}} p_{A_{0}}^{l_{n}}p_{A_{1}}^{{d-k-m-l}_{n}}\left( {1- \varepsilon}_{NC}^{A_{0}A_{y}} \right)^{l_{n}}\left( 1-\varepsilon_{NC}^{A_{1}A_{y}} \right)^{{d-k-m-l}_{n}} \right).$$

These two probabilities $P\left( Z_{d}=k | A=A_{y}, x=0 \right)$ and $P\left( Z_{d}=k | A=A_{y}, x=1 \right)$, were the remaining parts for Equation (3), which build the likelihood of the condom disposition model when taking attributes into account:

$$\prod_{i=1}^{n} P\left( Z_{d_{i}}=k_{i} \right|A_{i})=\prod_{i=1}^{n} P\left( Z_{d_{i}}=k_{i} | A=A_{i}, x=0 \right)\left( 1-p_{A_{i}} \right)+P\left( Z_{d_{i}}=k_{i} | A=A_{i}, x=1 \right)p_{A_{i}}.$$

For a model where attributes are considered but the non-condom probability when individuals with opposite dispositions meet are the same, we just need to set $\varepsilon_{NC}^{A_{0}A_{0}}=\varepsilon_{NC}^{A_{1}A_{0}} =\varepsilon_{NC}^{A_{0}A_{1}}=\varepsilon_{NC}^{A_{1}A_{1}}=\varepsilon$.

To allow females and males to have different distributions, we do similarly as in Hansson et al. [1] letting the dispositions of men and women be independent of each other, which leads to that the likelihood simply can be divided into a female part and a male part and multiply the two.

## S1.2. Recurring sex partners

In the following, we first explain the basis and the notation from Hansson et al [2] when we add the new parts regarding attributes this will be stated. Many aspects from the one-time partners disposition model hold here as well. Each individual draws a disposition independently, the disposition is drawn from a random variable $X\sim F$ which distribution $F$ we aim to infer. For one-time partners we used either 0 or 1 as disposition but we will for recurring sex partners use a disposition that can take all values between 0 and 1. This is because for one-time partners the outcome is binary (either a condom protected one-time sex act or a condomless one-time sex act) and therefore the models used a discrete distribution, For recurrent partners, however, the data consists of frequencies of condom use based on multiple sex acts within one or multiple partnerships, meaning that in a partnership condom could have been used for example half of the times, thus a continuous disposition distribution was used in order to enable a couple to have a mixture of condom outcomes. whereas for recurrent partners the data consists of frequencies of condom use based on multiple sex acts within one or multiple partnerships, thus a continuous disposition distribution was used. We will assume that the disposition distribution $F$ is a beta distribution (that is governed by the two positive parameters $\alpha$ and $\beta$). Denote the disposition of an individual $i$ by $x_{i}\in\left[ 0,1 \right]$, if in a pair of individual $i$ and individual $j$ their probability of not using a condom in a sex act is $y_{ij}=\sqrt{x_{i}x_{j}}$ (geometrical mean of the two dispositions). A difference from the one-time partners model is that two individuals now may generate several outcomes together since they have sex several times. Instead of the dispositions being binary (pro-condom or non-condom) and the outcome being binary (condom or no condom) as for the one-time partners, the condom dispositions for recurring partners range between 0-1 0 and 1.

Assume we have a sample of $n$ individuals, then let $d_{i}$ denote the number of recurring partners of individual $i$ and let $\vec{d}=(d_{1},\ldots,d_{n})$ be the vector of all individuals number of recurring partners. Among a sampled individual $i$ and its $j:th$ partner ($j=1,\ldots, d_{i}$), let the number of sex acts be denoted by $s_{ij}$ and the number of condomless sex acts by $k_{ij}$. Further, denote the vector of number of sex acts of individual $i$ and its $d_{i}$ partners by $\vec{s}_{i}=(s_{i1},\ldots,s_{id_{i}})$and similarly for the number of condomless sex acts $\vec{k}_{i}=(k_{i1},\ldots,k_{id_{i}})$, then for the whole sample we can express the data as the two vector of vectors $\vec{s}=(\vec{s}_{1},\ldots,\vec{s}_{n})$ and $\vec{k}=(\vec{k}_{1},\ldots,\vec{k}_{n})$. Let $K_{ij}$ be the random variable of the number of non-condom acts among a known number of sex acts. Note that. if we knew the disposition $x_{i},x_{j}$, and therefore would know $y_{ij}=\sqrt{x_{i}x_{j}}$, this would yield that $K_{ij}|y_{ij}\sim Bin(s_{ij},y_{ij})$. The likelihood is given by

$$L\left( \vec{k} \right|\vec{d},\vec{s})=\prod_{i=1}^{n} L\left( \vec{k}_{i} \right|d_{i},\vec{s}_{i})=\prod_{i=1}^{n} P\left( K_{i1}=k_{i1},\ldots,K_{id_{i}}=k_{id_{i}} \right|d_{i},\vec{s}_{i}).$$

The likelihood of the homogeneous case where everyone has the same disposition (each act have the same probability of being without condom) is given in [2]. The probability to observe individual $i's$ number of non-condom acts $P\left( K_{i1}=k_{i1},\ldots,K_{id_{i}}=k_{id_{i}} \right|d_{i},\vec{s}_{i})$ can be found by conditioning on first the disposition $x_{i}$ of individual $i$, since then the number of non-condom acts with the different partners will be independent. We will use the parameters ($\alpha_{i},\beta_{i}$) for the sampled individual $i$, to separate sampled individuals from its $d_{i}$ partners, enabling us to have different disposition distributions. Equation (5) in [2] and the derivations after gives that:

$$P\left( K_{i1}=k_{i1},\ldots,K_{id_{i}}=k_{id_{i}} \right|d_{i},\vec{s}_{i})=\int_{0}^{1} f_{X_{i}}\left( x_{i} \right)\prod_{j=1}^{d_{i}} P\left( K_{ij}=k_{ij} \right|s_{ij},x_{i}) dx_{i}=$$

$$= \int_{0}^{1} \frac{1}{B\left( \alpha_{i},\beta_{i} \right)}x_{i}^{\alpha_{i}-1}\left( 1-x_{i} \right)^{\beta_{i}-1}\prod_{j=1}^{d_{i}} P\left( K_{ij}=k_{ij} \right|s_{ij},x_{i}) dx_{i} ,$$

Where $B\left( \alpha_{i},\beta_{i} \right)$ is the beta function. The remaining part, $P\left( K_{ij}=k_{ij} \right|s_{ij},x_{i})$, the probability to observe $k_{ij}$ non-condom acts of individual $i$ with its partner $j=1,\ldots,d_{i}$, given the disposition $x_{i}$ and the number of sex acts $s_{ij}$ between them, is derived in [2]:

$$P\left( K_{ij}=k_{ij} \right|s_{ij},x_{i})=\frac{\left( \begin{matrix} s_{ij} \\ k_{ij} \end{matrix} \right)}{B\left( \alpha_{j},\beta_{j} \right)}\int_{0}^{1} {\sqrt{tx_{i}}}^{k_{ij}}\left( 1-\sqrt{tx_{i}} \right)^{s_{ij}-k_{ij}}t^{\alpha_{j}-1}\left( 1-t \right)^{\beta_{j}-1} dt. [4]$$

This gives us all parts for the likelihood when attributes is not yet taken into account.

*Incorporating psychosocial variables*

To include attributes, we assume that there are two different non-condom disposition distributions, $F_{A_{0}}$ and $F_{A_{1}},$ which both are beta distributed but with different parameters, $F_{A_{0}}\sim(\alpha_{A_{0}},\beta_{A_{0}})$ and $F_{A_{1}}\sim(\alpha_{A_{1}},\beta_{A_{1}})$. First, to find the likelihood when attributes are included, we need the attributes of all sampled individuals (seen as data and known):

$$L\left( \vec{k} \right|\vec{d},\vec{s},\vec{A} )=\prod_{i=1}^{n} L\left( \vec{k}_{i} \right|d_{i},\vec{s}_{i}, A_{i})=\prod_{i=1}^{n} \int_{0}^{1} \frac{1}{B\left( \alpha_{A_{i}},\beta_{A_{i}} \right)}x_{i}^{\alpha_{A_{i}}-1}\left( 1-x_{i} \right)^{\beta_{A_{i}}-1}\prod_{j=1}^{d_{i}} P\left( K_{ij}=k_{ij} \right|s_{ij},x_{i},A_{i}) dx_{i} ,.$$

To find $P\left( K_{ij}=k_{ij} \right|s_{ij},x_{i},A_{i})$we need to condition on the attribute of the $d_{i}$ partners (this is unknown).

$$P\left( K_{ij}=k_{ij} \right|s_{ij},x_{i},A_{i})= P\left( K_{ij}=k_{ij} \right|s_{ij},x_{i},A_{i},A_{j}=A_{0})q_{A_{0}}+P\left( K_{ij}=k_{ij} \right|s_{ij},x_{i},A_{i},A_{j}=A_{1})q_{A_{1}},$$

where

$$P\left( K_{ij}=k_{ij} \right|s_{ij},x_{i},A_{i},A_{j})= \frac{\left( \begin{matrix} s_{ij} \\ k_{ij} \end{matrix} \right)}{B\left( \alpha_{A_{j}},\beta_{A_{j}} \right)}\int_{0}^{1} {\sqrt{tx_{i}}}^{k_{ij}}\left( 1-\sqrt{tx_{i}} \right)^{s_{ij}-k_{ij}}t^{\alpha_{A_{j}}-1}\left( 1-t \right)^{\beta_{A_{j}}-1} dt.$$

All parts for the likelihood with attributes are therefore explained and can be put together.

# Table S1. Overview of all models estimating condom dispositions and probabilities in one-time partners.

| Models allowing the attributes to only estimate the distribution of condom dispositions | | | |  |  |  | Models allowing the attributes to also influence the condom probabilities | | | | | |
| --- | --- | --- | --- | --- | --- | --- | --- | --- | --- | --- | --- | --- |
|  | LogL | AIC | Proportion of individuals with a pro- condom disposition | Proportion of individuals with a non-condom disposition | Condom probability between opposite dispositions |  | LogL | AIC | Condom probability between opposite dispositions |  |  |  |
| Pro-con-neutral model | -185.0 | 376.0 | 0.45 | 0.27 | 0.01 |  |  |  |  |  |  |  |
| Pro-con model | -185.0 | 374.0 | 0.70 | 0.30 | 0.03 |  |  |  |  |  |  |  |
| Pro-con model including sex |  |  |  |  |  |  |  |  | PC male + NC female | NC male + PC female |  |  |
|  | -184.6 | 377.3 |  |  | 0.02 |  | -184.6 | 377.3 | 0.091 | 0.003 |  |  |
| Males |  |  | 0.68 | 0.32 |  |  |  |  |  |  |  |  |
| Females |  |  | 0.72 | 0.28 |  |  |  |  |  |  |  |  |
| Model including attitudes |  |  |  |  |  |  |  |  | NC moderately positive + PC moderately positive | NC strongly positive + PC moderately positive | NC moderately positive + PC strongly positive | NC strongly positive + PC strongly positive |
|  | -173.5 | 353.0 |  |  | 0.06 |  | -172.9 | 357.9 | 0.27 | 1 | 0 | 0.45 |
| Moderately positive |  |  | 0.54 | 0.46 |  |  |  |  |  |  |  |  |
| Strongly positive |  |  | 0.96 | 0.04 |  |  |  |  |  |  |  |  |
| Model including norms |  |  |  |  |  |  |  |  | NC moderately positive + PC moderately positive | NC strongly positive + PC moderately positive | NC moderately positive + PC strongly positive | NC strongly positive + PC strongly positive |
|  | -181.8 | 369.6 |  |  | 0.03 |  | -182.4 | 376.8 | 0 | 0.63 | 0.14 | 0 |
| Moderately positive |  |  | 0.60 | 0.40 |  |  |  |  |  |  |  |  |
| Strongly positive |  |  | 0.82 | 0.18 |  |  |  |  |  |  |  |  |
| Model including self-efficacy |  |  |  |  |  |  |  |  | NC lower + PC lower | NC higher + PC lower | NC lower + PC higher | NC higher + PC higher |
|  | -184.5 | 375.0 |  |  | 0.01 |  | -183.6 | 379.2 | 0 | 0.22 | 0 | 0.17 |
| Lower |  |  | 0.65 | 0.35 |  |  |  |  |  |  |  |  |
| Higher |  |  | 0.74 | 0.26 |  |  |  |  |  |  |  |  |
| Model including negotiation self-efficacy |  |  |  |  |  |  |  |  | NC lower + PC lower | NC higher + PC lower | NC lower + PC higher | NC higher + PC higher |
|  | -183.2 | 372.4 |  |  | 0.01 |  | -182.7 | 377.1 | 0.04 | 0.45 | 0 | 0 |
| Lower |  |  | 0.63 | 0.37 |  |  |  |  |  |  |  |  |
| Higher |  |  | 0.79 | 0.21 |  |  |  |  |  |  |  |  |

*NC=Non-condom disposition, PC=Pro-condom disposition

# S2. Questionnaire, translated from Dutch

**Thank you for participating in this study!**

**What is this questionnaire about?**

This questionnaire is about sex, sexual behavior, and the use of condoms. We are asking you and many other young people to answer these questions.

**Who can participate?**

Young people in the Netherlands aged 16 to 24 years old.

It doesn’t matter whether you have had sex or not.

**Why are we asking these questions?**

We want to know how young people think about condoms and when they do or don’t use them. This way, we can provide better information to young people or help them with their problems.

**Do you have to participate?**

Participation in this study is voluntary. You can stop participating at any time if you no longer wish to continue.

**What happens to your answers?**

Your information will be treated confidentially. The researchers cannot see who you are.

**How do you participate?**

- Answer the questions and use the arrows at the bottom to go to the next question.
- You cannot go back to previous questions.
- There are no wrong answers. It’s about what you think or do.
- At the top of the page, you can see how far along you are.

As a thank-you, we are giving away x number of €25 VVV gift cards! You can use these at, for example, bol.com or Thuisbezorgd.

More information about the study and your privacy can be found and downloaded [here].

*Link to the information sheet*

Have questions or experiencing any problems while filling out the questionnaire? Contact the researchers via email (email blinded).

**Online consent form for the study: How do young people think about condom use?**

I have read and understood the ‘Information sheet for participants’ for this study. I understand that I can stop participating at any time. The researchers will use the data I have provided until that point. I consent to participate in this study.

That means I give permission for:

1. Participation in the study.
2. Collecting data about who I am (gender, age, and background).
3. Collecting data about what I do (condom use and sexual activities).
4. Collecting data about my sexual health (STI tests and diagnoses).

o	Yes

o	No

I agree that my research data may be stored in Data Archiving and Networked Services (DANS) and on data.rivm.nl, so that other researchers can view them. The data will not reveal who I am. If I do not consent, I can still participate in the study.

o	Yes

o	No

Now, there are some general questions about you.

1. **How old are you?**

____________________

2. **Were you born as a boy or a girl? (This refers to how your parents registered you with the municipality.)**

- Boy
- Girl

3. **How do you identify? (Your feeling may differ from how you were born.)**

- A boy
- A girl
- Somewhere between a boy and a girl
- Both a boy and a girl
- Neither a boy nor a girl
- Other, namely ...
- I don’t know (yet)

If 2 and 3 indicate that the participant is not cisgender:

4. **Have you had surgery to change your genitalia?**

- Yes, I was born with a vagina and now have a penis.
- No, I was born with a vagina and still have a vagina.
- Yes, I was born with a penis and now have a vagina.
- No, I was born with a penis and still have a penis.

5. **How do you see yourself?**

- Heterosexual
- Homosexual or gay
- Lesbian or gay
- Bisexual
- Pansexual
- Queer
- Asexual
- Other, namely ...
- I don’t know
- I’d rather not say

6. **What is the highest level of education you have completed?**

- Elementary school
- Special education
- VMBO
- MBO 1
- MBO 2, 3, or 4
- HAVO
- VWO
- HBO
- University

7. **In which country were you born?**

- The Netherlands
- Turkey
- Another European country
- Morocco
- Another African country
- Suriname
- The Antilles
- Another South/Central American country
- A North American country
- Indonesia
- Japan
- Another Asian country
- Australia or New Zealand
- I don’t know

8. **In which country was your mother born?**

(Same country options as above)

9. **In which country was your father born?**

(Same country options as above)

10. **With which country or culture do you feel the most connected?**

(Same country options as above)

11. **Is there another country or culture with which you feel connected?**

- Yes
- No

If 11 = Yes:

**12. With which second country or culture do you feel the most connected?**

(Same country options as above)

**Now we’ll ask a few questions about whether you’ve ever had sex.**

13. **Have you ever had vaginal sex (penis in vagina)?**

- Yes
- No
- I’d rather not say

14. **With how many people have you had vaginal sex (penis in vagina) so far?**

Provide an estimate if unsure.

__________________

15. **How old were you when you first had vaginal sex?**

This refers to the first partner you had vaginal sex with after the age of 13. Provide an estimate if unsure.

__________________

**The next questions are about you, your opinions regarding condoms, sex, and health.**

**We will also ask questions about STIs.**

**What is an STI?**

STI stands for sexually transmitted infection. Another word for this is venereal disease.

An example of an STI is chlamydia.

**16. How do you feel about the following statements**?

Strongly Disagree	Strongly Agree

1	2	3	4	5

I have a lot of self-confidence.

I trust that I can use a condom.

I trust that I can convince a partner to use a condom.

I plan to use a condom when I have sex.

**17. How do you feel about the following statements?**

Strongly Disagree	Strongly Agree

1	2	3	4	5

When I use a condom, I trust that I am protected against STIs.

Using condoms is an effective way to avoid STIs.

When I use a condom, I know for sure that I am protected from STIs.

I would feel bad if I got an STI.

**18. People can get an STI. Thinking about how you live now, how likely do you think it is that you will get an STI?**

Provide a percentage between 0% (absolutely no chance of getting an STI) and 100% (very high chance of getting an STI).

_________________

**19. How do you feel about the following statements?**

Strongly Disagree	Strongly Agree

1	2	3	4	5

People who are important to me think I should use a condom when I have sex.

People who are important to me use a condom when they have sex.

I feel comfortable using condoms.

People like me (e.g., classmates and friends) feel comfortable using condoms.

**20. How do you feel about the following statements?**

Strongly Disagree	Strongly Agree

1	2	3	4	5

I am motivated to stay healthy.

I think it’s important to use a condom during sex.

I pay less than average attention to my health.

My health is important to me.

I am motivated to protect myself during sex.

I think it’s important to make sure I don’t get an STI.

**21. How do you feel about the following statements?**

Strongly Disagree	Strongly Agree

1	2	3	4	5

I use condoms automatically.

I use condoms during sex without consciously remembering to.

I use condoms during sex without thinking about it.

I’m already using a condom before I even realize it.

In the heat of the moment, I often forget to use a condom.

If I use alcohol or drugs, I forget to use a condom.

**22. How do you feel about using condoms?**

Bad	Good

1	2	3	4	5

Unpleasant	Pleasant

1	2	3	4	5

Not comfortable	Comfortable

1	2	3	4	5

Has disadvantages	Has advantages

1	2	3	4	5

Irresponsible	Responsible

1	2	3	4	5

**The next questions describe a hypothetical scenario. Imagine it is about you and a hypothetical partner.**

Scenario for female:

You are single and you encounter an attractive acquaintance, Fynn, in a bar. You chat for a while and both notice that it's a pleasant conversation. Fynn is funny and genuinely interested in what you have to say. You spend the whole evening together. At the end, he walks you home. You ask if he wants to come in, and he says yes. After chatting and kissing on the couch for a while, it's clear that you both want to have sex with each other. [sentence that varies for each condition]

Scenario for male:

You are single and you encounter an attractive acquaintance, Lynn, in a bar. You chat for a while and both notice that it's a pleasant conversation. Lynn is funny and genuinely interested in what you have to say. You spend the whole evening together. At the end, she walks you home. You ask if she wants to come in, and she says yes. After chatting and kissing on the couch for a while, it's clear that you both want to have sex with each other. [sentence that varies for each condition]

- Attitudes:
  - Positive: Fynn/Lynn says that he/she finds it important and pleasant to use condoms and asks, "what shall we do?"
  - Negative: Fynn/Lynn says that he/she finds it unimportant and unpleasant to use condoms and asks, "what shall we do?"
- Norms:
  - Positive: Fynn/Lynn says that his/her friends have sex with condoms and asks, "what shall we do?
  - Negative: Fynn/Lynn says that his/her friends have sex without condoms and asks, "what shall we do?"
- Risk behavior:
  - Low: Fynn/Lynn has only had one long-term relationship before you and asks, "what shall we do?"
  - High: Fynn/Lynn has had multiple short-term relationships before you and asks, "what shall we do?"

**23. If I were in this situation, I would use a condom.**

Highly unlikely	Highly likely

1	2	3	4	5

**24. I think Fynn/Lynn would want to use a condom.**

Highly unlikely	Highly likely

1	2	3	4	5

**Now we’ll ask about your sex life and condom use.**

**25. Have you had vaginal sex (penis in vagina) in the past 12 months?**

- Yes
- No
- I’d rather not say

If 25 = No, the survey ends.

**26. In the past 12 months, have you had vaginal sex (penis in vagina)?**

- Yes
- No
- I’d rather not say

END OF SURVEY IF QUESTION 25 = NO

**27. With how many different partners have you had vaginal sex (penis in vagina) in the past 12 months?**

Provide an estimate if you’re not sure.

________________

**27. With how many of these partners did you have sex for the first time in the past 12 months?**

Provide an estimate if you’re not sure.

________________

**28. How often do you use condoms during vaginal sex?**

- Never
- Mostly not
- Sometimes yes, sometimes no
- Mostly yes
- Always

It’s possible that you have engaged in different types of sex in the past 12 months, or that you’ve had sex with someone of the same gender. For the following questions, we are only asking about people with whom you’ve had vaginal sex (penis in vagina).

**29. Partner 1**

Think of the partner you most recently had vaginal sex with. For the following questions, you can use the first letter(s) of their name or a nickname.

First letter(s) or nickname: _________________________

**30. How many times have you had vaginal sex with Partner 1?**

- 1 time
- More than once

If 30 = 1 time:

**31. Did you and Partner 1 use a condom that time?**

- Yes
- No
- I don’t know

If 30 = More than once:

**32. How long did you have vaginal sex with Partner 1?**

- Less than 1 month
- 1 month or longer

**33. How long did you have vaginal sex with Partner 1?**

______ weeks/months

**34. On average, how often did you and Partner 1 have vaginal sex per week/month?**

Provide an estimate if you’re not sure.

________ times per week/month

**35. How often did you and Partner 1 use condoms during the period when you were having sex?**

- Never
- Mostly not
- Sometimes yes, sometimes no
- Mostly yes
- Always

**36. Do you think you and Partner 1 will have sex again in the future?**

- Yes
- No
- I don’t know

*If respondents had ≥ 2 sexual partners in the past 12 months:

The questions about partners (29–35) will be repeated for each partner, up to a maximum of 10 partners. If a respondent reports having more than 1 partner, the same set of questions will be asked for each partner individually.

The survey stops after 5 partners, even if you had 6 or more partners.*

**Now we’ll ask some questions about STIs. After that, the survey is complete.**

**37. Have you ever had an STI test?**

- Yes, in the past 12 months
- Yes, more than 12 months ago
- No

If 37 = Yes:

**38. Have you ever had an STI? If yes, which STI?**

- No, I’ve never had an STI
- Yes, I had chlamydia
- Yes, I had another STI
- Yes, I had chlamydia and another STI
- I don’t know
- If the respondent reports having chlamydia:

**39. When did you have chlamydia?**

- In the past 12 months
- More than 12 months ago
- Both in the past 12 months and before (you’ve had chlamydia multiple times)

**Thank you for completing the questionnaire!**

**Would you like more information about love, sex, or STIs? Visit [sense.info].**

**As a thank-you, you can enter a prize draw. You can win a €25 VVV gift card!**

**With this gift card, you can purchase items at, for example, bol.com or Thuisbezorgd.**

**For the prize draw, we need your email address. The researchers will not see which email address corresponds to which answers.**

**I would like to participate in the prize draw and therefore consent to my email address being used.**

- Yes
- No

# S3. STROBE checklist

STROBE Statement—Checklist of items that should be included in reports of ***cross-sectional studies***

|  | Item No | Recommendation | Reported on page # |
| --- | --- | --- | --- |
| **Title and abstract** | 1 | (*a*) Indicate the study’s design with a commonly used term in the title or the abstract | 1 |
|  |  | (*b*) Provide in the abstract an informative and balanced summary of what was done and what was found | 1-2 |
| **Introduction** |  |  |  |
| Background/rationale | 2 | Explain the scientific background and rationale for the investigation being reported | 3-4 |
| Objectives | 3 | State specific objectives, including any prespecified hypotheses | 4 |
| **Methods** |  |  |  |
| Study design | 4 | Present key elements of study design early in the paper | 4-9 |
| Setting | 5 | Describe the setting, locations, and relevant dates, including periods of recruitment, exposure, follow-up, and data collection | 4 |
| Participants | 6 | (*a*) Give the eligibility criteria, and the sources and methods of selection of participants | 4 |
| Variables | 7 | Clearly define all outcomes, exposures, predictors, potential confounders, and effect modifiers. Give diagnostic criteria, if applicable | 4-7 |
| Data sources/ measurement | 8* | For each variable of interest, give sources of data and details of methods of assessment (measurement). Describe comparability of assessment methods if there is more than one group | 4-7 |
| Bias | 9 | Describe any efforts to address potential sources of bias | 4 |
| Study size | 10 | Explain how the study size was arrived at | 9 |
| Quantitative variables | 11 | Explain how quantitative variables were handled in the analyses. If applicable, describe which groupings were chosen and why | 4-7 |
| Statistical methods | 12 | (*a*) Describe all statistical methods, including those used to control for confounding | 7-9, supplement |
|  |  | (*b*) Describe any methods used to examine subgroups and interactions | 7-9, supplement |
|  |  | (*c*) Explain how missing data were addressed | 9 |
|  |  | (*d*) If applicable, describe analytical methods taking account of sampling strategy | NA |
|  |  | (*e*) Describe any sensitivity analyses | NA |
| **Results** |  |  |  |
| Participants | 13* | (a) Report numbers of individuals at each stage of study—eg numbers potentially eligible, examined for eligibility, confirmed eligible, included in the study, completing follow-up, and analysed | 9 |
|  |  | (b) Give reasons for non-participation at each stage | NA |
|  |  | (c) Consider use of a flow diagram | NA |
| Descriptive data | 14* | (a) Give characteristics of study participants (eg demographic, clinical, social) and information on exposures and potential confounders | 9, table 1 |
|  |  | (b) Indicate number of participants with missing data for each variable of interest |  |
| Outcome data | 15* | Report numbers of outcome events or summary measures | 8-10 |
| Main results | 16 | (*a*) Give unadjusted estimates and, if applicable, confounder-adjusted estimates and their precision (eg, 95% confidence interval). Make clear which confounders were adjusted for and why they were included | 9-10 |
|  |  | (*b*) Report category boundaries when continuous variables were categorized | NA |
|  |  | (*c*) If relevant, consider translating estimates of relative risk into absolute risk for a meaningful time period | NA |
| Other analyses | 17 | Report other analyses done—eg analyses of subgroups and interactions, and sensitivity analyses | NA |
| **Discussion** |  |  |  |
| Key results | 18 | Summarise key results with reference to study objectives | 11 |
| Limitations | 19 | Discuss limitations of the study, taking into account sources of potential bias or imprecision. Discuss both direction and magnitude of any potential bias | 11-12 |
| Interpretation | 20 | Give a cautious overall interpretation of results considering objectives, limitations, multiplicity of analyses, results from similar studies, and other relevant evidence | 10-14 |
| Generalisability | 21 | Discuss the generalisability (external validity) of the study results | 14 |
| **Other information** |  |  |  |
| Funding | 22 | Give the source of funding and the role of the funders for the present study and, if applicable, for the original study on which the present article is based | 16 |

*Give information separately for exposed and unexposed groups.

**Note:** An Explanation and Elaboration article discusses each checklist item and gives methodological background and published examples of transparent reporting. The STROBE checklist is best used in conjunction with this article (freely available on the Web sites of PLoS Medicine at http://www.plosmedicine.org/, Annals of Internal Medicine at http://www.annals.org/, and Epidemiology at http://www.epidem.com/). Information on the STROBE Initiative is available at www.strobe-statement.org.

# References

1.	Hansson D, Fridlund V, Stenqvist K, Britton T, Liljeros F. Inferring individual sexual action dispositions from egocentric network data on dyadic sexual outcomes. PLOS ONE. 2018;13(11):e0207116.

2.	Hansson D, Strömdahl S. Estimating individual action dispositions using binary and frequency egocentric sexual network data. Statistica Neerlandica. 2020;74(3):242-60.
